# Where are the trans masculinities in the SUS? Sociodemographic and access profile of trans men and transmasculine individuals linked to the Transgender Outpatient Clinic in Porto Alegre, Rio Grande do Sul state, Brazil, 2019-2021

**DOI:** 10.1590/S2237-96222024v33e2024133.especial.en

**Published:** 2024-11-04

**Authors:** Guilherme Lamperti Thomazi, Gabriela Tizianel Aguilar, Andrei Fernandes da Rocha, Nathália Pacífico de Carvalho, Matheus Neves

**Affiliations:** 1 Universidade de São Paulo, Faculdade de Saúde Pública, São Paulo, SP, Brazil; 2 Aids Healthcare Foundation Brazil, Porto Alegre, RS, Brazil; 3 Ministério da Saúde, Secretaria de Atenção Especializada, Brasília, DF, Brazil; 4 Universidade Federal de Minas Gerais, Faculdade de Medicina, Belo Horizonte, MG, Brazil; 5 Universidade Federal do Rio Grande do Sul, Departamento de Odontologia Preventiva e Social, Porto Alegre, RS, Brazil

**Keywords:** Servicios de Salud para las Personas Transgénero, Hombres Transgénero, Atención Primaria de Salud, Estudios Transversales, Equidad, Accesibilidad a los Servicios de Salud, Health Services for Transgender People, Trans Men, Primary Health Care, Cross-sectional Studies, Equity, Access to Health Services

## Abstract

**Objective:**

To analyze the sociodemographic and access profile of trans men and transmasculine individuals linked to the Transgender Outpatient Clinic in Porto Alegre, capital city of the state of Rio Grande do Sul.

**Methods:**

This was a descriptive cross-sectional study based on data from users registered with the service between 2019 and 2021.

**Results:**

Of the 418 people included, 384 (91.2%) identified as trans men and 34 (8.8%) as transmasculine individuals. The majority were of White race/skin color (77.9%) and 16.4% had a right to name and gender rectification. Scheduled appointments were the predominant mode of access (84.0%). Among the trans men, 188 (49.0%) had utilized primary healthcare services prior to receiving care at the outpatient clinic.

**Conclusion:**

The users were predominantly young, White, with higher levels of education, and were minimally engaged in the formal labor market. The existence of a service staffed with qualified and sensitized professionals can enhance the access of this population to the Brazilian National Health System (*Sistema Único de Saúde* – SUS).

## Study contributions

**Main results:**

Less than half of trans men had used primary healthcare services prior to receiving care at the Transgender Outpatient Clinic. Users are predominantly young, White, with higher levels of education and are minimally engaged in the formal labor market.

**Implications for services:**

The results can serve as a basis for structuring and improving healthcare services and policies for trans men and transmasculine individuals within the SUS. It also shows that specialized and sensitized professionals can expand access to healthcare.

**Perspectives:**

It is essential to develop comprehensive studies (both qualitative and quantitative) that evaluate the barriers to accessing the SUS faced by trans men and transmasculine individuals, as well as feasible ways to reduce them.

## RESUMEN

**Objetivo:**

analizar el perfil sociodemográfico y el acceso de hombres trans y transmasculinos vinculados al Ambulatorio Trans de Porto Alegre entre 2019 y 2021.

**Métodos:**

Se utilizó un diseño transversal y descriptivo con datos secundarios de los usuarios registrados en el servicio entre 2019 y 2021.

**Resulta:**

Del total, 384 (91,2%) se identificaron como hombres trans y 34 (8,8%) como transmasculinos. La mayoría son de blancos (77,9%). La minoría (16,4%) tuvo acceso a rectificación de nombre y sexo. La cita previa fue la forma más habitual de acceso (84,0%). Entre los hombres trans, 188 (49,0%) habían visitado la atención primaria antes de la consulta.

**Conclusión:**

La mayoría de los usuarios son jóvenes blancos, mayor nivel educativo y baja participación en el mercado laboral formal. La existencia de un servicio con profesionales cualificados y sensibilizados facilita el acceso de esta población al SUS.

## INTRODUCTION

The Brazilian National Health System (*Sistema Único de Saúde* - SUS) plays a fundamental role in the lives of the Brazilian population, with universality, comprehensiveness and equity as its pillars.¹ Addressing the health needs of all social groups is crucial. Trans people and transvestites face transphobia characterized by the dehumanization and marginalization of their existence. This form of violence is pervasive in their lives, interconnected with other forms of discrimination and negatively impacting their access to healthcare.^2,3^ Addressing healthcare demands and developing and improving public policies are essential.^4^

The first Brazilian health policy specific to trans people is Ordinance No. 1,707, issued on August 18, 2008, by the Ministry of Health, which established the Transsexualization Process within the SUS. Initially, five registered centers performed hormone therapy and body modification surgeries in the country, without specifically addressing trans men and transmasculine individuals.^5,6^ Three years later, the National LGBT Comprehensive Health Policy was established and, in 2013, the Transsexualization Process was redefined to include specific procedures for trans men.^4^.^7^

The implementation of these policies is incipient, often based on a pathologizing view of trans experiences, considering them as individuals with treatable mental disorders and limited agency. With the emergence of the HIV/AIDS epidemic, health care actions for this population focused on sexual health, alcohol and substance abuse, and body modifications, often neglecting a comprehensive approach to the individual.^8^

Due to the inadequate consideration of gender identity by the Census and other official surveys, such as the National Household Sample Survey and the National Health Survey, there is a lack of statistics on this population in the country. It is estimated that around 2% of the Brazilian population identify as trans, transvestite and non-binary people.^9^ The group of trans men and transmasculine individuals is even more marginalized, absent in official data and rarely addressed in health research and other fields of knowledge.^6^.^10^

This context expresses the significant challenge in analyzing the profile of these populations and planning effective public policies to combat discrimination. Although transmasculine identity is not new, the scarcity of studies on the health of this population is evident in Brazil and Latin America.^10^ Taking into consideration the historical processes of invisibilization affecting trans men and transmasculine individuals, this article aims to analyze the sociodemographic profile of access and care at the Transgender Outpatient Clinic for trans men and transmasculine individuals, in Porto Alegre, state of Rio Grande do Sul.

## METHODS

### Study design and setting

This was a descriptive cross-sectional study based on the analysis of data from trans men and transmasculine individuals registered with the Transgender Outpatient Clinic in Porto Alegre, between 2019 and 2021.

Porto Alegre is the capital and largest city in the state of Rio Grande do Sul, with a population of 1,332,845 inhabitants.^11^ Despite this significant population, until 2019 there was only one service providing specialized care for trans people and transvestite in the municipality: the Transdisciplinary Gender Identity Program at the Hospital de Clínicas de Porto Alegre, where the waiting time for the first appointment could exceed 12 months.^6^.^12^

Due to the demand for qualified, humanized and accessible care for this population in the city, the Transgender Outpatient Clinic was inaugurated on August 7, 2019, coordinated by the Municipal Health Department of Porto Alegre. Its operation, location, opening hours and services offered were planned and developed in conjunction with the transgender and transvestite social movements in the city. Initially, the Transgender Outpatient Clinic was open every Wednesday, from 6 pm to 10 pm, at a Primary Health Center in the city center, with access through spontaneous demand and prior appointment via WhatsApp. As demand increased, service days and hours were extended, and it now opens Monday to Friday, from 1 pm to 7 pm. This is an open-door SUS service providing primary care procedures and services to trans people and transvestites residing in Porto Alegre, state of Rio Grande do Sul. The unique aspects of the Transgender Outpatient Clinic include a sensitized team regarding health issues of trans people and transvestites, providing harmonization and the free dispensing of hormones.^12^

### Study population

The study population is comprised of trans men and transmasculine individuals registered at the Transgender Outpatient Clinic in Porto Alegre, according to the following inclusion criteria: (i) identifying as a trans man or transmasculine individual and (ii) having been welcomed at the Transgender Outpatient Clinic in Porto Alegre and having had a scheduled appointment, not necessarily performed, since the service opened (August 17, 2019) until two years later (August 17, 2021). Individuals with incomplete or inconsistent data in the consulted sources were excluded.

### Data source

The database containing people registered at the Transgender Outpatient Clinic was made available by the service coordination on September 2, 2021. The primary variable in the database, Gender Identity, was categorized as trans men, trans women, transvestites, non-binary people, people with another gender identity and people without information. Taking into account that the service database did not include the category “transmasculine” and that this is not a homogeneous group, the researchers established criteria to categorize data on this population, taking into account the processes of historical invisibilization to which they have been subject to. Thus, in this study, the transmasculine category includes people who were assigned female at birth and who identify with neutral and/or masculine pronouns and have a non-binary gender identity. The trans men category includes individuals assigned female at birth who identify with this gender identity.

In order to select the sample included in this study, the data available in the database were requalified. In addition, information from electronic medical records in the e-SUS system was used. The e-SUS APS information system (e-SUS *Atenção Primária*), proposed by the Ministry of Health, includes several modules; for this study the Electronic Citizen Medical Record was used. Porto Alegre was among the first capitals to adopt this system.

Health professionals record service progress in these electronic medical records, including gender self-identification, information requested from users at the start of care. Thus, information from both sources (service database and e -SUS) were manually combined to improve data completeness. Subsequently, the requalification process was carried out in two stages: (i) analysis of cases identified as “non-binary identities”, “other” or without a record (n = 108), which had their electronic medical records in the e-SUS analyzed and reclassified (34 transmasculine individuals; 18 trans men and 55 trans women and transvestites, the latter were not included); and (ii) review of the e-SUS medical records of all users identified as trans men (n = 515), leading to the exclusion of one duplicate record, two cases that were actually trans women, and five cases identifying as transfeminine.

### Study variables

In addition to the gender identity variable (trans men or transmasculine individuals), obtained and categorized as previously described, sociodemographic variables and variables related to access and care service were included in the study. The sociodemographic variables included were: self-declared race/skin color (White, Black, mixed-race, Asian and Indigenous), age group (5-15, 16-19, 20-29, 30-39, 40-49, 50+), schooling (incomplete elementary education, complete elementary education, incomplete high school, complete high school, incomplete higher education, complete higher education), formal employment (yes or no; “no” includes people who are unemployed, self-employed, or students) and name rectification (yes or no). For the analysis of parameters related to access and care, the following variables were included: mode of access (prior appointment via WhatsApp application or spontaneous demand), type of care (face-to-face or online), primary health care utilization (yes or not, corresponding to the use of any primary heathcare service in the 12 months prior to registering at the Transgender Outpatient Clinic, verified through the progress observed in the e-SUS electronic medical records from other health centers in the municipality, as this is the system used both in primary and specialized care in Porto Alegre).

All of these variables were obtained from the Transgender Outpatient Clinic service database, except for the prior linkage variable, obtained from the e-SUS electronic medical record.

### Analysis

Variables were presented using descriptive statistics (absolute value of numbers and absolute and relative frequencies), stratified by gender identity and race/skin color. The variables related to service attendance were presented stratified by gender identity and race/skin color. Data processing was performed using Excel 365.

### Ethical aspects

This study was approved by the Research Ethics Committee of the Universidade Federal do Rio Grande do Sul, through opinion No. 5.207.201, on 20/1/2022 (Ethical Approval Code - CAEE 53148521.9.0000.5347) and by the CEP of the Porto Alegre Municipal Health Department, through opinion No. 5.278.354, on 8/3/2022 (CAEE 53148521.9.3001.5338).

## RESULTS

A total of 418 people were included in this study, of whom 384 were trans men (91.2%) and 34 (8.8%) transmasculine individuals. The majority of the sample (78.5%) identified as White, aged 20 to 29 years (58.4%) and had complete high school or incomplete higher education (29.2% and 28.2%, respectively). Most of them did not have a formal job (69.4%) and had not had their name legally changed (84.9%). None of the transmasculine individuals in the sample had their name legally changed, while a small percentage (16.4%) of trans men had achieved this right (Table 1).

**Table 1:** Sociodemographic characteristics of trans men and transmasculine individuals registered at the Porto Alegre Transgender Outpatient Clinic, Brazil, 2019-2021 (n = 418)

Characteristic	Trans men n = 384		Transmasculine individuals n = 34		Total n = 418	
	n	%	n	%	n	%
Race/skin color						
White	299	77.9	29	85.3	328	78.5
Black	57	14.8	5	14.7	62	14.8
Mixed-race	25	6.5	0	0.0	25	6.0
Asian	3	0.8	0	0.0	3	0.7
Age range (years)						
5-15	14	3.7	2	5.9	16	3.8
16-19	58	15.1	3	8.8	61	14.6
20-29	221	57.6	23	67.7	244	58.4
30-39	70	18.2	5	14.7	75	17.9
40-49	15	3.9	1	2.9	16	3.8
≥50	6	1.6	0	0.0	6	1.4
Schooling						
Incomplete elementary education	39	10.2	2	5.9	41	9.8
Complete elementary education	13	3.4	0	0.0	13	3.1
Incomplete high school	90	23.4	4	11.8	64	15.3
Complete high school	115	30.0	7	20.6	122	29.2
Incomplete higher education	103	26.8	15	44.1	118	28.2
Complete higher education	24	6.3	6	17.7	30	7.2
Formal work						
Yes	122	31.8	6	17.7	128	30.6
No	262	68.2	28	82.4	290	69.4
Name legally changed						
Yes	63	16.4	0	0	63	15.1
No	321	83.6	34	100.0	355	84.9

The majority of participants (84.0%) accessed the Transgender Outpatient Clinic service through a scheduled appointment (Table 2). Of the total number of participants, 91.2% attended the service in person. It was found that less than half (47.6%) of the sample had used primary healthcare services in the previous 12 months. Among trans men, this percentage was 49.0%, while only 32.4% of transmasculine individuals showed evidence of prior linkage to primary care services (Table 2).

**Table 2:** Mode of access, type of care and use of primary healthcare services among trans men and transmasculine individuals registered at the Porto Alegre Transgender Outpatient Clinic, Brazil, 2019-2021 (n = 418)

Characteristic	Trans men (n = 384)		Transmasculine individuals (n = 34)		Total (n = 418)	
	n	%	n	%	n	%
Mode of access						
Scheduled appointment	318	82.8	33	97.1	351	84.0
Spontaneous demand	66	17.2	1	2.9	67	16.0
Type of care						
Face-to-face	355	92.4	26	76.5	381	91.2
Online	29	7.6	8	25.5	37	8.9
Use of primary healthcare						
Yes	188	49.0	11	32.4	199	47.6
No	196	51.0	23	67.7	219	52.4

## DISCUSSION

The trans men and transmasculine individuals who used the Porto Alegre Transgender Outpatient Clinic between 2019 and 2021 were predominantly, young, White, with higher level of education and had low formal labor market participation. The use of primary healthcare in the previous 12 months was rare, especially among those who identify as transmasculine individuals, indicating a significant gap between this group and primary healthcare services.

Despite the smaller proportion of transmasculine individuals in the sample compared to trans men, we deemed it is crucial to present stratified data to highlight the distinct identities within this population, thus addressing another form of invisibility faced by this community. Recognizing and documenting this emerging identity among youth is essential. To the best of author’s knowledge, this study represents the largest specific sample of trans men and transmasculine individuals within the SUS.

In a non-probabilistic national survey on transmasculinity based on 1,219 online responses, the authors concluded that the participant profile was predominantly comprised of young, White trans men and transmasculine individuals with access to higher education,^13^ results similar to those of this study. This profile contrasts with that of transgender people and transvestites who are predominantly Black, of low-income and engage in sex work – a group more likely to be victims of homicide.^14^

Our study identified a strong presence of young people accessing the service, consistent with the findings of the national survey on Brazilian transmasculinity, where 44% of respondents were aged 19 and 24 years and 36.6% were aged 25 and 34 years.^13^ This trend also aligns with data from a cohort study conducted at a clinic responsible for the care of 95% of trans people in the Netherlands between 1972 and 2015, where the average age of the 1,624 trans men who started follow-up at this service was 25 years old.^15^

A national study conducted in the United States using data from 2014 and 2015, reported that trans people are younger than the overall population. This conclusion is based on the results in which 300,000 adolescents aged 13 to 17 years are transgender, representing approximately 1.6% of the U.S. population.^16^ This percentage decreases to 0.6% among people over 18 years of age, totaling 1.3 million transgender adults, 38.5% of whom are trans men. In Brazil, there is currently no population-based study specifically addressing the sociodemographic characteristics of trans people. However, a group of researchers conducted a study across the five regions of the country and found that the average age of trans people in Brazil is 28.5 years old, compared to an average age of 42 years among cisgender adults.^9^ The same study showed that 96.3% of trans people in Brazil have only completed high school as their highest level of education, and just 3.7% have completed higher education. In contrast, the 2022 Demographic Census indicates that 41.5% of Brazilians aged 25 to 64 years did not complete high school, and 28% did not even have access to high school education.^17^

The findings of this study align more closely with census data, showing that 28% of participants did not complete high school. However, due to the method of data collection and the inclusion of people under 18 years of age, it is unclear whether this figure reflects ongoing education at the time of data collection or results from dropout. Nonetheless, it is noteworthy that the literature identifies dropout as a potential cause of lower education level in this population, driven by factors such as the refusal to recognize social names and use of gender-appropriate restroom, verbal abuse and humiliation, which contribute to marginalization and, consequently, increased social vulnerability. ^14,18,19^

Another significant finding of this study is that a minority of trans men have had their names legally changed on official documents. Although delayed, the National Council of Justice Provision No. 73 of June 28, 2018 (which provides for the procedures and documents required for this process) granted trans people the right to a legal name, a right enjoyed by all cisgender people. However, 17 mandatory documents are required for the registration. These documents are not free of charge, combined with the fact that less than half of the study participants have formal employment, this could explain the low percentage of people who exercised this right.^20^ Additionally, it is mandatory to change the gender marker to the “opposite” sex, that is, it is not possible, unless by legal means, an option that welcomes non-binary people.

Despite the clinic’s expansion of operating days, online prior scheduling remained the primary mode of access to the Porto Alegre Transgender Outpatient Clinic, utilized by the majority of users. Furthermore, during the COVID-19 pandemic, both online consultations via video calls and face-to-face visits were offered. The high number of people accessing the service through prior scheduled-appointment underscores the centrality of digital media in the lives of young people. This finding is consistent with data from the 2022 National Household Sample Survey (*Pesquisa Nacional por Amostra de Domicílio* – PNAD), which shows that 93.5% of young people aged 20 to 24 years and 94.8% of those aged 25 to 29 years have a cell phone for personal use.^21^ This indicates that social media could be an effective tool for expanding access to healthcare.

Regarding healthcare access, 47.6% of trans men included in the sample had used primary healthcare services before accessing the Transgender Outpatient Clinic, a figure that drops to 32.4% among transmasculine individuals. Both figures are significantly low, with transmasculine individuals being less than half. Data from the 2019 National Health Survey (*Pesquisa Nacional de Saúde* - PNS) indicate that 69.9% of people assigned female at birth aged 18 years and older accessed primary healthcare services in the six months prior to the interview.^22^ This reduced access may be attributed to the various barriers that must be overcome to access healthcare services. Access is not limited to entering the healthcare system, but also encompasses how individuals use the healthcare services and how their specific needs are taken into account, considering the geographic, economic and functional issues.^23,24^ In this study, access was measured based on the use of primary healthcare services 12 months prior to registration at the Transgender Outpatient Clinic. One limitation of this measurement is that some healthcare professionals may not update their consultations due to structural restrictions, such as limited access to computers and stable internet connections. In addition to the barriers faced by the general population, trans people and transvestites face an additional obstacle: transphobia.

Transphobia manifests in healthcare settings in various ways, including disrespect for social names and chosen pronouns, lack of knowledge about specific healthcare needs and the absence of fields for social names and/or gender identity in medical records and systems.^1,2,25,26^ Furthermore, trans men and transmasculine individuals are often perceived as outsiders in healthcare settings, subjected to uneasy stares.^27^ As a result of these negative experiences, many of them are reluctant to affirm their gender identity and avoid using healthcare services.^28^

While barriers faced by trans women and transvestites in the SUS have already been identified, there is a lack of evidence regarding trans men and transmasculine individuals.^6,8,29,30^ Although some obstacles are shared, there are specificities that are rarely addressed, such as the difficulty trans men face in accessing gynecological care or cytology tests after name/gender rectification, given that the healthcare system blocks these services for males. Trans men and transmasculine individuals also face unique experiences, including issues related to uterus, risk of unintended pregnancy, sexual violence, access to abortion in cases provided by law, contraception, prenatal care that is appropriated for trans experiences.^31^

One of the strengths of the Transgender Outpatient Clinic, which has led to increased use of the SUS by trans men and transmasculine individuals, refers to the opportunity for users to feel that they belong to the service. This process involves encouraging the participation of the social movement and civil society since the service’s inception, with weekly support group meetings, workshops on various topics and local health council meetings.^12^

The limitations of this study stem from its descriptive nature, which prevents inferences about changes over time and comparisons between groups based on different outcomes. The convenience sampling method based on people’s admission to the service during a specific period, limits the possibility of extrapolating the results obtained. By including only trans men and transmasculine individuals who accessed the Porto Alegre Transgender Outpatient Clinic, this sample does not necessarily represent the reality of all trans men and transmasculine individuals, especially those who would not be accessing healthcare services via the SUS. Despite these limitations, presenting data on this often, invisible group, is valuable for fostering discussions and, consequently, for the improvement and implementation of existing policies. This study provides data on the users of a specialized Transgender Outpatient Clinic in Porto Alegre, staffed by healthcare professionals trained and sensitized to meet the needs of the transgender and transvestite populations. The data include information about who the trans men and transmasculine individuals registered with the service are, as well as their prior use of primary healthcare services.

The existence of a service that invests in the training of healthcare professionals increases access to the SUS for this population. It is also essential to understand who is not accessing healthcare services in order to develop strategies that ensure the right to health for all people. Although the outpatient clinic offers advantages for access by this group, it faces restrictions regarding opening hours and the number of professionals on the team, suggesting that expansion is necessary to meet demand. However, the existence of specialized services for trans people should not justify the denial or restriction of access to other healthcare services, underscoring the need for continuous training and sensitization of healthcare professionals and managers to provide comprehensive and equitable care for the transgender population.
