# Supplementary material for: Where are the trans masculinities in the SUS? Sociodemographic and access profile of trans men and transmasculine individuals linked to the Transgender Outpatient Clinic in Porto Alegre, Rio Grande do Sul state, Brazil, 2019-2021
Source: Epidemiol Serv Saude. 2024 Nov 4;33(spe1):e2024133. doi: 10.1590/S2237-96222024v33e2024133.especial.en (PMC11534072; doi:10.1590/S2237-96222024v33e2024133.especial.en)
Supplement: Translation

# Translation: Onde estão as transmasculinidades no SUS? Perfil sociodemográfico e de acesso de homens trans e transmasculinos vinculados ao Ambulatório Trans de Porto Alegre, Rio Grande do Sul, 2019-2021

**DOI:** 10.1590/S2237-96222024v33e2024133.especial.pt

**Authors:** Guilherme Lamperti Thomazi, Gabriela Tizianel Aguilar, Andrei Fernandes da Rocha, Nathália Pacífico de Carvalho, Matheus Neves

**Affiliations:** 1 Universidade de São Paulo, Faculdade de Saúde Pública, São Paulo, SP, Brasil; 2 Aids Healthcare Foundation Brasil, Porto Alegre, RS, Brasil; 3 Ministério da Saúde, Secretaria de Atenção Especializada, Brasília, DF, Brasil; 4 Universidade Federal de Minas Gerais, Faculdade de Medicina, Belo Horizonte, MG, Brasil; 5 Universidade Federal do Rio Grande do Sul, Departamento de Odontologia Preventiva e Social, Porto Alegre, RS, Brasil

**Keywords:** Serviços de Saúde para Pessoas Transgênero, Homens Trans, Atenção Primária à Saúde, Estudos Transversais, Equidade, Acesso aos Serviços de Saúde

## Abstract

**Objetivo:**

Analisar o perfil sociodemográfico e de acesso de homens trans e transmasculinos vinculados ao Ambulatório Trans de Porto Alegre.

**Métodos:**

Trata-se de estudo transversal descritivo com base em dados dos usuários cadastrados no serviço entre 2019 e 2021.

**Resultados:**

Das 418 pessoas incluídas, 384 (91,2%) se identificaram como homens trans e 34 (8,8%) como pessoas transmasculinas. A maioria era branca (77,9%) e 16,4% tiveram acesso ao direito de retificação do nome e gênero. O agendamento foi a forma majoritária de ingresso (84,0%). No grupo de homens trans, 188 (49,0%) frequentaram a atenção básica antes do ambulatório.

**Conclusão:**

Os usuários eram majoritariamente brancos, jovens, com mais anos de estudo e pouco inseridos no mercado de trabalho formal. A existência de um serviço com profissionais qualificados e sensibilizados em relação a pessoas trans amplia o acesso dessa população ao SUS.

## Contribuições do estudo

**Principais resultados:**

Menos da metade dos homens trans tinha utilizado um serviço de atenção primária antes de ingressar no Ambulatório Trans. Os usuários eram majoritariamente brancos, jovens, com mais anos de estudo e pouco inseridos no mercado de trabalho formal.

**Implicações para os serviços:**

Os resultados podem servir de base para estruturação e qualificação de serviços e políticas de saúde para homens trans e transmasculinos no SUS. Também mostra que profissionais especializados e sensibilizados podem ampliar o acesso à saúde.

**Perspectivas:**

É fundamental o desenvolvimento de estudos abrangentes (tanto qualitativos como quantitativos) que avaliem as barreiras de acesso ao SUS enfrentadas por homens trans e transmasculinos e formas factíveis de diminuí-las.

## INTRODUÇÃO

O Sistema Único de Saúde (SUS) tem papel fundamental na vida da população brasileira, tendo a universalidade, integralidade e equidade como pilares.¹ Considerar os impactos no acolhimento das necessidades em saúde para todos os grupos sociais se faz necessário. As pessoas trans e travestis enfrentam a transfobia, marcada pela desumanização e subalternização de suas existências. Essa forma de violência é uma constante na vida dessa população, interligando-se com outras formas de discriminação e impactando negativamente seu acesso à saúde.^2,3^ O acolhimento de demandas em saúde se mostra relevante, assim como a criação e qualificação de políticas públicas.^4^

A primeira política brasileira de saúde específica para pessoas trans é a Portaria nº 1.707, de 18 de agosto de 2008, do Ministério da Saúde, que instituiu o Processo Transexualizador no SUS. Inicialmente, cinco centros cadastrados realizavam a hormonização e as cirurgias de modificação corporal no país, sem ainda abordarem especificamente homens trans e transmasculinos.^5,6^ Três anos depois, foi instituída a Política Nacional de Saúde Integral LGBT e, em 2013, o Processo Transexualizador foi redefinido para incluir procedimentos específicos para homens trans.^4,7^

A implementação dessas políticas é incipiente, muitas vezes baseando-se em visão patologizante de vivências trans, considerando as pessoas trans portadoras de transtornos mentais tratáveis e com pouca agência. Com o surgimento da epidemia de HIV/aids, as ações de cuidado para essa população focaram saúde sexual, uso abusivo de álcool e outras drogas, e modificações corporais, frequentemente negligenciando uma abordagem integral da pessoa.^8^

Devido à inadequada consideração da identidade de gênero pelo Censo Demográfico e outras pesquisas oficiais, como a Pesquisa Nacional por Amostra de Domicílios (PNAD) e a Pesquisa Nacional de Saúde (PNS), faltam estatísticas sobre essa população no país. Estima-se que cerca de 2% da população brasileira identifiquem-se enquanto pessoas trans, travestis e não binárias.^9^ O grupo de homens trans e transmasculinos é ainda mais invisibilizado, ausente nos dados oficiais e pouco abordado na produção científica da saúde e em outros campos do conhecimento.^6,10^

Esse contexto expressa o significativo desafio para as análises de perfil dessas populações e o planejamento de políticas públicas eficazes contra a discriminação. Embora as identidades transmasculinas não sejam novas, a escassez de estudos sobre a saúde dessa população é evidente no Brasil e na América Latina.^10^ Considerando os processos históricos de invisibilização que afetam homens trans e transmasculinos, este artigo objetiva analisar o perfil sociodemográfico de acesso e atendimento ao Ambulatório Trans de Porto Alegre para homens trans e transmasculinos.

## MÉTODOS

### Desenho do estudo e contexto 

Trata-se de estudo transversal descritivo a partir da análise de dados de homens trans e transmasculinos cadastrados no Ambulatório Trans da cidade de Porto Alegre, entre os anos de 2019 e 2021. 

Porto Alegre é a capital e maior cidade do estado do Rio Grande do Sul, contando com 1.332.845 habitantes.^11^ Mesmo com esse significativo contingente populacional, até o ano de 2019 existia apenas um serviço que prestava atendimento especializado para pessoas trans e travestis do município, o Programa Transdisciplinar de Identidade de Gênero do Hospital de Clínicas de Porto Alegre, no qual o tempo de espera para a primeira consulta poderia ser superior a 12 meses.^6,12^

A partir da demanda por atendimento qualificado, humanizado e acessível para essa população no município, o Ambulatório Trans foi inaugurado em 7 de agosto de 2019, e é coordenado pela Secretaria Municipal de Saúde de Porto Alegre. Seu funcionamento, localização, horários e atendimentos ofertados foram pensados e construídos em conjunto com os movimentos sociais de pessoas trans e travestis do município. No primeiro momento, o Ambulatório Trans funcionava todas as quartas-feiras, das 18 às 22 horas, em uma Unidade Básica de Saúde no centro da cidade e tinha como forma de ingresso a demanda espontânea e agendamento prévio via aplicativo WhatsApp. Após o aumento da procura, foram ampliados os dias e horários de atendimento, funcionando o Ambulatório de segunda a sexta-feira, das 13 às 19 horas. Trata-se de um serviço do SUS, porta aberta, que oferta procedimentos e atendimentos usuais da atenção básica, tendo como população adscrita as pessoas trans e travestis com residência em Porto Alegre/RS. Os diferenciais do Ambulatório Trans são a sensibilização da equipe sobre as questões de saúde de pessoas trans e travestis, a realização de harmonização e a dispensação gratuita de hormônios.^12^

### População do estudo

A população do estudo é composta de homens trans e transmasculinos cadastrados no Ambulatório Trans de Porto Alegre, de acordo com os seguintes critérios de inclusão: (i) identificar-se enquanto homem trans ou transmasculino e (ii) ter passado pelo acolhimento no Ambulatório Trans de Porto Alegre e ter tido consulta agendada, não necessariamente realizada, desde a inauguração do serviço (17 de agosto de 2019) até dois anos após (17 de agosto de 2021). Foram excluídas aquelas pessoas cujos dados presentes nas fontes de dados consultadas estavam incompletos ou inconsistentes.

### Fonte dos dados

O banco de dados contendo as pessoas cadastradas no Ambulatório Trans foi disponibilizado pela coordenação do serviço no dia 2 de setembro de 2021. A variável principal do banco, “Identidade de Gênero”, era categorizada em homens trans, mulheres trans, travestis, pessoas não binárias, pessoas com outra identidade de gênero e pessoas sem informação. Tendo em vista que o banco do serviço não apresentava a categoria “transmasculinos” e que esse não é um grupo homogêneo, as(os) pesquisadoras(es) estabeleceram critérios para categorizar dados sobre essa população, levando em consideração os processos de invisibilização histórica aos quais foi submetida. Assim, neste trabalho, na categoria transmasculinos foram incluídas as pessoas que tiveram o sexo feminino atribuído ao nascimento e que se identificam com pronomes neutros e/ou masculinos e com identidade de gênero não binária. A categoria de homens trans inclui pessoas que tiveram o sexo feminino atribuído ao nascimento e que se identificam com essa identidade de gênero. 

Desse modo, para a seleção da amostra incluída neste estudo, foi realizado um processo de requalificação dos dados disponíveis no referido banco de dados. Foram consideradas, de forma complementar, informações contidas em prontuários eletrônicos do sistema e-SUS. O sistema de informação e-SUS APS foi proposto pelo Ministério da Saúde e conta com diversos módulos; dentro deles, o utilizado para este estudo foi o Prontuário Eletrônico do Cidadão, e Porto Alegre foi uma das primeiras capitais a adotar seu uso. 

As(os) profissionais de saúde registram as evoluções dos atendimentos nesses prontuários eletrônicos, incluindo a informação de identidade de gênero autodeclarada, que é solicitada aos usuários no início do atendimento. Assim, as informações de ambas as fontes (banco de dados do serviço e e-SUS) foram manualmente combinadas para melhoria da completude das informações. Após essa etapa, iniciou-se o processo de requalificação, realizado em duas etapas: (i) análise dos casos identificados como “identidades não binárias”, “outro” ou sem registro (n = 108), os quais tiveram seus prontuários eletrônicos no e-SUS analisados e, a partir disso, foram reclassificados (34 transmasculinos; 18 homens trans e 55 mulheres trans e travestis, estas últimas não incluídas); e (ii) revisão do prontuário no e-SUS de todos os usuários identificados como homens trans (n = 515), que levou à exclusão de um caso de registro duplicado, dois casos que eram, na verdade, mulheres trans, e cinco casos que se identificavam enquanto transfemininas. 

### Variáveis do estudo

Além da variável identidade de gênero (homens trans ou transmasculinos), obtida e categorizada conforme descrito anteriormente, foram incluídas no estudo variáveis sociodemográficas e variáveis relativas ao acesso e ao atendimento no serviço. As variáveis sociodemográficas incluídas foram: raça/cor da pele autodeclarada (branco, preto, pardo, amarelo e indígena), faixa etária (5-15, 16-19, 20-29, 30-39, 40-49, 50+), escolaridade (ensino fundamental incompleto, ensino fundamental completo, ensino médio incompleto, ensino médio completo, ensino superior incompleto, ensino superior completo), trabalho formal (sim ou não; o “não” contempla casos de pessoas desempregadas, autônomas ou que são estudantes) e nome retificado (sim ou não). Para a análise de parâmetros relacionados ao acesso e ao atendimento, as seguintes variáveis foram incluídas: forma de acesso (agendamento prévio por meio do aplicativo WhatsApp ou demanda espontânea), tipo de atendimento (presencial ou *online*), utilização da atenção básica (sim ou não, que corresponde à utilização de algum serviço da atenção básica nos 12 meses anteriores ao cadastro no Ambulatório Trans, verificada pela presença de evolução em prontuário eletrônico do e-SUS de outras unidades de saúde do município, visto que esse é o sistema utilizado tanto na atenção básica como na especializada de Porto Alegre). 

Todas essas variáveis foram obtidas a partir do banco de dados do serviço do Ambulatório Trans, com exceção da variável vínculo prévio, obtida a partir do prontuário eletrônico do e-SUS. 

### Análises

As variáveis foram apresentadas utilizando-se estatísticas descritivas (números absolutos e frequências absolutas e relativas), estratificadas por identidade de gênero. As variáveis relativas ao atendimento no serviço foram apresentadas estratificadas por identidade de gênero e por raça/cor da pele. O programa Excel 365 foi utilizado para o processamento dos dados.

### Aspectos éticos

O presente estudo foi aprovado pelo Comitê de Ética em Pesquisa da Universidade Federal do Rio Grande do Sul, por meio do parecer 5.207.201 de 20/1/2022 (Código de Aprovação Ética de Estudos – CAEE 53148521.9.0000.5347) e pelo CEP da Secretaria Municipal de Saúde de Porto Alegre, por meio do parecer 5.278.354, de 8/3/2022 (CAEE 53148521.9.3001.5338).

## RESULTADOS

Um total de 418 pessoas foram incluídas neste estudo, das quais 384 eram homens trans (91,2%) e 34 (8,8%) pessoas transmaculinas. A maioria da amostra (78,5%) declarou-se branca e tinha entre 20 e 29 anos (58,4%) e ensino médio completo ou ensino superior incompleto (29,2% e 28,2%, respectivamente). A maior parte não estava vinculada a um trabalho formal (69,4%) e não teve o nome retificado (84,9%). Nenhum dos transmasculinos da amostra teve seu nome retificado, enquanto um percentual pequeno (16,4%) dos homens trans conseguiram esse direito (Tabela 1).

**Tabela 1:** Características sociodemográficas de homens trans e transmasculinos cadastrados no Ambulatório Trans de Porto Alegre, 2019-2021 (n = 418)

Característica	Homens trans n = 384		Transmasculinos n = 34		Total n = 418	
	**n**	**%**	**n**	**%**	**n**	**%**
**Raça/cor da pele**						
Branca	299	77,9	29	85,3	328	78,5
Preta	57	14,8	5	14,7	62	14,8
Parda	25	6,5	0	0,0	25	6,0
Amarela	3	0,8	0	0,0	3	0,7
**Faixa etária (anos)**						
5-15	14	3,7	2	5,9	16	3,8
16-19	58	15,1	3	8,8	61	14,6
20-29	221	57,6	23	67,7	244	58,4
30-39	70	18,2	5	14,7	75	17,9
40-49	15	3,9	1	2,9	16	3,8
≥ 50	6	1,6	0	0,0	6	1,4
**Escolaridade**						
Ensino fundamental incompleto	39	10,2	2	5,9	41	9,8
Ensino fundamental completo	13	3,4	0	0,0	13	3,1
Ensino médio incompleto	90	23,4	4	11,8	64	15,3
Ensino médio completo	115	30,0	7	20,6	122	29,2
Ensino superior incompleto	103	26,8	15	44,1	118	28,2
Ensino superior completo	24	6,3	6	17,7	30	7,2
**Trabalho formal**						
Sim	122	31,8	6	17,7	128	30,6
Não	262	68,2	28	82,4	290	69,4
**Nome retificado**						
Sim	63	16,4	0	0	63	15,1
Não	321	83,6	34	100,0	355	84,9

A maioria dos participantes (84,0%) acessou o serviço do Ambulatório Trans por meio de agendamento (Tabela 2). Do total de participantes, 91,2% compareceram presencialmente ao serviço para o atendimento. Verificou-se que menos da metade (47,6%) da amostra fez uso da atenção básica nos 12 meses anteriores. Entre os homens trans, essa porcentagem foi de 49,0%, enquanto para os transmasculinos apenas 32,4% apresentaram indícios dessa vinculação prévia à atenção básica (Tabela 2).

**Tabela 2:** Forma de acesso, tipo de atendimento e utilização da atenção básica para homens trans e transmasculinos cadastrados no Ambulatório Trans de Porto Alegre, 2019-2021 (n = 418)

Característica	Homens trans (n =384)		Trasmasculinos (n = 34)		Total (n = 418)	
	**n**	**%**	**n**	**%**	**n**	**%**
**Forma de acesso**						
Agendamento	318	82,8	33	97,1	351	84,0
Demanda espontânea	66	17,2	1	2,9	67	16,0
**Tipo de atendimento**						
Presencial	355	92,4	26	76,5	381	91,2
*Online*	29	7,6	8	25,5	37	8,9
**Utilização da atenção básica**						
Sim	188	49,0	11	32,4	199	47,6
Não	196	51,0	23	67,7	219	52,4

## DISCUSSÃO

Os homens trans e transmasculinos usuários do Ambulatório Trans de Porto Alegre, entre 2019 e 2021, eram majoritariamente brancos, jovens, mais escolarizadas e pouco inseridos no mercado de trabalho formal. A utilização da atenção primária, nos 12 meses anteriores, foi pouco frequente, especialmente para aqueles que se identificavam enquanto transmasculinos, evidenciando uma distância desse grupo da atenção básica. 

Mesmo que a porcentagem da amostra composta por transmasculinos seja consideravelmente menor que a de homens trans, julgamos crucial a apresentação estratificada de quem são essas pessoas, como uma medida para combater mais uma forma de invisibilidade enfrentada por essa comunidade, sendo necessária a demarcação da existência dessa identidade emergente e presente entre a juventude. Este artigo investigou a maior amostra específica de homens trans e transmasculinos no SUS, de acordo com o melhor conhecimento dos autores. 

Em mapeamento nacional sobre transmasculinidades não probabilístico, baseado em 1.219 respostas a formulário *online*, os autores concluíram que o perfil dos participantes é composto majoritariamente por homens trans e transmasculinos jovens, brancos e com acesso ao ensino superior,^13^ resultados similares aos deste estudo. Esse perfil contrasta com o de pessoas trans e travestis que são assassinadas, composto majoritariamente por pessoas negras, de baixa renda e profissionais do sexo.^14^

Em nosso estudo, foi constatada forte presença de jovens que acessam o serviço, dado congruente com os obtidos no mapeamento das transmasculinidades brasileiras, em que 44% dos respondentes tinham entre 19 e 24 anos e 36,6% entre 25 e 34 anos.^13^ Essa também é uma realidade presente em uma coorte realizada na clínica responsável pelo atendimento de 95% das pessoas trans da Holanda entre os anos de 1972 e 2015, em que 25 é a idade média dos 1.624 homens trans que iniciaram o acompanhamento nesse serviço.^15^

Um estudo nacional realizado nos Estados Unidos, com dados de 2014 e 2015, afirma que pessoas trans são mais jovens do que a população em geral. Essa conclusão se dá a partir dos resultados, em que 300 mil adolescentes entre 13 e 17 anos são trans, representando cerca de 1,6% da população estadunidense.^16^ Essa porcentagem cai para 0,6% das pessoas com mais de 18 anos, totalizando um montante de 1,3 milhão de adultos trans, sendo 38,5% homens trans. Já no Brasil, atualmente, não existe um estudo de base populacional específico sobre as características sociodemográficas de pessoas trans. Com isso em mente, um grupo de pesquisadores, após conduzir um estudo nas cinco regiões do país, descobriu que a idade média das pessoas trans no Brasil é de 28,5 anos, em contraste com a idade média de 42 anos entre adultos cisgêneros.^9^ Esse mesmo estudo aponta que 96,3% das pessoas trans no Brasil têm como mais alta formação o ensino médio, e apenas 3,7% completaram o ensino superior. Em contraste, o Censo Demográfico de 2022 aponta que 41,5% dos brasileiros entre 25 e 64 anos não completaram o ensino médio, e a porcentagem daqueles que nem conseguiram acessar o ensino médio é de 28%.^17^

Os achados neste trabalho se aproximam mais dos resultados do censo, apresentando que 28% não finalizaram o ensino médio. No entanto, devido ao método de coleta da variável e à existência de pessoas com menos de 18 anos, não podemos afirmar se esse número decorre de o curso ainda estar em andamento, no momento da coleta, ou se é resultado de abandono. Mesmo assim, é importante mencionar que a literatura acusa o abandono como uma possível causa de menor escolaridade nessa população, seja pela negativa do nome social e uso do banheiro de acordo com o gênero autodeclarado, seja por xingamentos e humilhações, o que resulta em sua marginalização e, consequentemente, maior vulnerabilização pela sociedade.^14,18,19^

Outro importante resultado deste estudo é de que a minoria dos homens trans têm o nome retificado nos seus documentos. Mesmo que tardio, o Provimento da Corregedoria Nacional de Justiça nº 73, de 28 de junho de 2018, que dispõe sobre a forma e os documentos necessários para a realização desse processo, trouxe para as pessoas trans a possibilidade de um direito a que todas as pessoas cis têm – o nome. Entretanto, são necessários 17 documentos obrigatórios para que a averbação aconteça. Documentos esses que não são gratuitos, o que, somado ao fato de que menos da metade dos usuários deste estudo têm emprego formal, pode explicar a baixa porcentagem de pessoas que usufruíram desse direito.^20^ Outro ponto a ser levantado é que é obrigatória a mudança para o sexo “oposto”, ou seja, não é possível, a menos que seja por via judicial, opção que acolha pessoas não binárias.

Mesmo após a expansão de dias de funcionamento, o agendamento prévio *online* continuou como método principal de entrada no Ambulatório Trans de Porto Alegre, sendo utilizado pela maioria dos usuários. Além disso, durante a pandemia de covid-19, foram realizados atendimentos *online* por videochamadas, além dos presenciais, que foram mantidos. O alto número de pessoas que ingressaram no serviço por meio de agendamento anterior ao comparecimento no Ambulatório mostra a centralidade que as mídias digitais têm na vida dos jovens. Essa informação é consonante com os dados da PNAD 2022, que mostram que 93,5% dos jovens entre 20 e 24 anos e 94,8% daqueles com idade entre 25 e 29 anos têm aparelho celular de uso pessoal.^21^ Isso indica que o uso das redes sociais pode ser profícuo para ampliar o acesso.

Tangenciando o acesso à saúde, dos homens trans incluídos na amostra, 47,6% utilizaram um serviço de atenção básica antes de ingressar no Ambulatório Trans, número que cai para 32,4% entre os transmasculinos. Ambos os números são inferiores, sendo o de transmasculinos menos da metade. Os dados da PNS 2019 apontam que 69,9% das pessoas com sexo feminino ao nascimento, maiores de 18 anos, utilizaram algum serviço da atenção primária nos seis meses antes da entrevista.^22^ Esse acesso reduzido pode ser explicado pelas variadas barreiras que precisam ser superadas para o acesso a um serviço de saúde. O acesso não se restringe à entrada no serviço de saúde, mas também sobre como a pessoa o utiliza e como suas especificidades são acolhidas, considerando-se a questão geográfica, econômica e funcional.^23,24^ Neste trabalho, o acesso foi medido a partir do uso de serviços de saúde da atenção básica 12 meses antes do cadastro no Ambulatório Trans. Um dos limites dessa mensuração é que alguns profissionais podem não realizar evoluções de seus atendimentos devido a restrições de ordem estrutural, como a disponibilidade de computadores e de sinal estável de internet. Além das barreiras que permeiam o acesso da população em geral, pessoas trans e travestis enfrentam um obstáculo a mais, a transfobia. 

A transfobia se materializa na saúde de diversas formas: no desrespeito ao nome social e pronomes de escolha, desconhecimento de especificidades em saúde e inexistência dos campos nome social e/ou identidade de gênero em prontuários e sistemas diversos.^1,2,25,26^ Além disso, homens trans e transmasculinos são vistos como corpos estranhos e não pertencentes no serviço, sendo alvos de olhares inquietos.^27^ Como consequências dessas experiências negativas, temos a relutância em assumir sua identidade e não utilização de serviços de saúde.^28^

Barreiras enfrentadas por mulheres trans e travestis no SUS já foram identificadas, com escassez de evidências sobre homens trans e transmasculinos.^6,8,29,30^ Mesmo que alguns obstáculos sejam compartilhados, existem especificidades pouco abordadas, como a dificuldade que homens trans que retificam o nome/gênero encontram ao buscar profissional ginecologista ou exames citopatológicos, tendo em vista que o sistema bloqueia essa especialidade para o “sexo” masculino. Homens trans e transmasculinos estão sujeitos a outras vivências que permeiam o cotidiano de pessoas, como risco de gestação indesejada, violência sexual, acesso ao aborto em casos previsto por lei, contracepção, pré-natal adequado à realidade e vivências trans.^31^

Uma das potências do Ambulatório Trans, que resulta no aumento da utilização do SUS por parte de homens trans e transmasculinos, refere-se à oportunização das pessoas usuárias de se sentirem pertencentes ao serviço. Esse processo envolve incentivos à participação do movimento social e da sociedade civil desde a estruturação do serviço, com encontros semanais de grupos de convivência, oficinas de diversos temas e reuniões do conselho local de saúde.^12^

As limitações deste estudo decorrem de seu caráter descritivo, que impede inferências sobre mudanças ao longo do tempo e comparações entre grupos, a depender do desfecho analisado. O processo de amostragem por conveniência (pela admissão das pessoas no serviço) e o período são fatores que comprometem a possibilidade de extrapolação dos resultados obtidos. Ao incluir apenas homens trans e transmasculinos que acessaram o Ambulatório Trans de Porto Alegre, essa amostra não representa, necessariamente, a realidade de todos os homens trans e transmasculinos, especialmente daqueles que não estariam acessando serviços de saúde no SUS. Mesmo com essas limitações, a apresentação de dados referentes a um grupo bastante invisibilizado é profícua ao possibilitar discussões e, por consequência, qualificação e implementação de políticas já existentes. Este trabalho apresenta dados relacionados aos usuários de um Ambulatório Trans em Porto Alegre especializado no atendimento à população trans e travesti, com profissionais capacitados e sensibilizados para atender a suas demandas. Os dados abordam informações sobre quem são os homens trans e transmasculinos cadastrados no serviço, além da existência de uso prévio de serviços da atenção básica. 

A existência de um serviço que investe na qualificação de profissionais amplia o acesso dessa população ao SUS. É essencial compreender também quem não acessa os serviços de saúde, para o desenvolvimento de estratégias que garantam o direito à saúde para todas as pessoas. O ambulatório, apesar das vantagens que apresenta para acesso desse grupo, tem restrições quanto ao horário e ao número de profissionais na equipe, sugerindo-se a expansão para melhor atendimento da demanda. Contudo, a existência de serviços específicos para pessoas trans não deve justificar a negação ou restrição do acesso a outros serviços de saúde, sendo necessária a sensibilização e capacitação contínua dos profissionais e gestores para atender de forma integral e equitativa a população trans.
